# Received anti-VEGF therapy in a patient with CRAO sparing the CLRA with subretinal fluid: A case report

**DOI:** 10.1097/MD.0000000000031204

**Published:** 2022-11-11

**Authors:** Yu-Jie Jia, Hong-Bo Liu, Yuan Qin, Jing-Hui Liu, Fa-Li Jia, Han Zhang, Jia-Hao Li, Ying-Jun Li

**Affiliations:** a Department of Ophthalmology, Fuyang People’s Hospital of Anhui Medical University, Fuyang, Anhui, China; b Department of Emergency Medicine, Fuyang People’s Hospital of Anhui Medical University, Fuyang, Anhui, China; c Department of Ophthalmology, Affiliated Hospital of Yanbian University, Yanji, Jilin, China.

**Keywords:** acute central retinal artery, anti-VEGF, cherry red spot, cilioretinal artery, subretinal fluid

## Abstract

Central retinal artery occlusion (CRAO) sparing the cilioretinal artery (CLRA) with severe subretinal fluid and non-characteristic cherry-red spot is uncommon. In the present case, the patient received an intravitreal injection of anti- vascular endothelial growth factor, which is very rare.

**Patient concerns:**

A 59-year-old man underwent vitrectomy of the left eye for diabetic retinopathy and vitreous hemorrhage. Six months after the operation, the patient presented with sudden painless visual-acuity decline in his left eye and was diagnosed with CRAO; his best corrected visual acuity was weak light perception. Whole retinal edema was seen on the fundus, and macular gray retinal opacification was present without a characteristic cherry-red spot. Optical coherence tomography revealed subretinal fluid (SRF) in the papillomacular bundle and inner retinal thickening. Fundus fluorescein angiography confirmed that the central retinal artery was not filled at 40 seconds and that the CLRA supplied a part of the macular area. Eight months after the second intravitreal injection of ranibizumab, Optical coherence tomography showed a significant reduction in inner retinal hyperreflectivity and the thickness of the nasal macular retina. The SRF was clearly absorbed, and the visual acuity improved to 1.1 logMAR units.

**Diagnosis:**

Atypical CRAO.

**Interventions:**

The patient was treated with intravitreal injection of anti-VEGF in his left eye. The thickness of the nasal macular retina decreased.

**Outcomes:**

The SRF was clearly absorbed, and the patient’s visual acuity significantly improved.

**Lessons:**

When CRAO occurs in patients with diabetic retinopathy sparing the CLRA, the non-characteristic cherry-red spot may be due to macular inner retinal edema, SRF and other factors. According to the patient’s condition, anti-vascular endothelial growth factor can be administered as appropriate to inhibit choroidal neovascularization, reduce SRF in the macular retina, and save some vision.

## 1. Introduction

Central retinal artery occlusion (CRAO) is a critical and urgent ophthalmologic disease that can cause a sharp decline in or even permanent loss of visual function within a short time and thus must be diagnosed and treated urgently.^[1]^ CRAO is characterized by whitening of the posterior retina, a cherry-red spot, a pale optic disc, and narrowing of the arterial diameter.^[2,3]^ Risk factors for CRAO include hypertension, carotid atherosclerosis, structural cardiac pathology, coronary heart disease, cerebro vascular accident, and diabetes mellitus.^[4–6]^ When the central retinal artery is blocked, the cilioretinal artery (CLRA) supplies the macula and nearby retinal blood flow in approximately 11% of patients so that the papillomacular bundle and macula are not damaged, and some of the central vision can be saved.^[7]^ This is the first report of a patient undergoing vitrectomy for diabetic retinopathy (DR) and vitreous hemorrhage who experienced CRAO 6 months after the operation and exhibited subretinal fluid (SRF) in the papillomacular bundle, but without a cherry-red spot. After intravitreal injection of anti-vascular endothelial growth factor (VEGF), the SRF was absorbed, and the patient’s vision improved.

## 2. Case report

**Figure 1.**
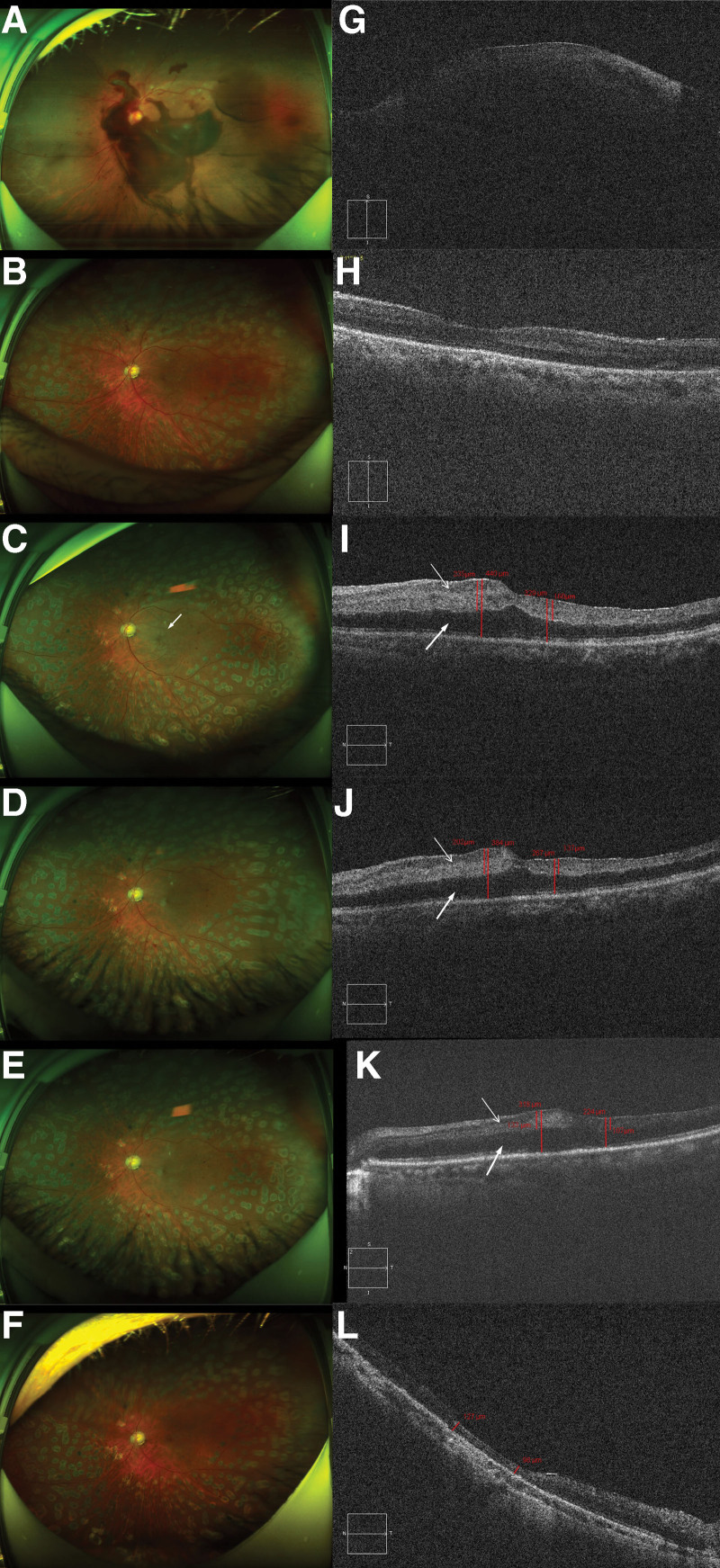
At the first visit. (A) Color fundus photo of the left eye showing vitreous hemorrhage, extensive patchy hemorrhage around the optic disc, and a large amount of neovascularization at the posterior pole. (G) OCT showed that the macular area was blurred because of preretinal hemorrhage. Two months after the surgery. (B) The fundus showed a smooth retina and a full retinal laser spot. (H) OCT showed a normal retinal morphology in the macular area. Occurrence of CRAO 2 days later. (C) The fundus shows whole retinal edema and gray retinal opacification in the macula without a cherry-red spot (white arrow). (I) OCT shows inner retinal edema and hyperreflectivity (thin arrow), nasal macular neurosensory retinal detachment, and SRF in the papillomacular bundle (thick arrow). Additionally, the inner layer on the nasal side was thinner than that on the nasal side. Two weeks after intravitreal ranibizumab injection. (D) Fundus image. (J) OCT revealed reduced retinal edema in the macular area and hyperreflectivity of the inner retinal layers (thin arrow) but no significant changes in subretinal fluid accumulation (thick arrow). Two weeks after the second intravitreal ranibizumab injection. (E) Fundus image. (K) OCT revealed reduced edema and hyperreflectivity of the macular inner retina (thin arrow), and a part of the residual SRF was observed (thick arrow). Six months after the second intravitreal ranibizumab injection (F) Fundus image. (L) OCT revealed that the nasal macular retina had become thin and atrophic, and the SRF was completely absorbed. CRAO = Central retinal artery occlusion, OCT = optical coherence tomography, SRF = subretinal fluid.

A 59-year-old male patient had a 3-year history of hypertension, a 40-year history of hepatitis B, and a 15-year history of diabetes treated with subcutaneous insulin injection. In February 2021, the patient underwent surgical procedures including phacoemulsification and intraocular lens (IOL) implantation combined with vitrectomy, membrane delamination, and panretinal photocoagulation in the left eye for DR complicated by vitreous hemorrhage (Fig. 1A and G). Two months after the operation, the patient’s intraocular pressure was 12 mm Hg, and the IOL was placed. The fundus showed a smooth retina and whole retinal laser spot (Fig. 1B). Optical coherence tomography (OCT) showed a normal macular shape (Fig. 1H).

**Figure 2.**
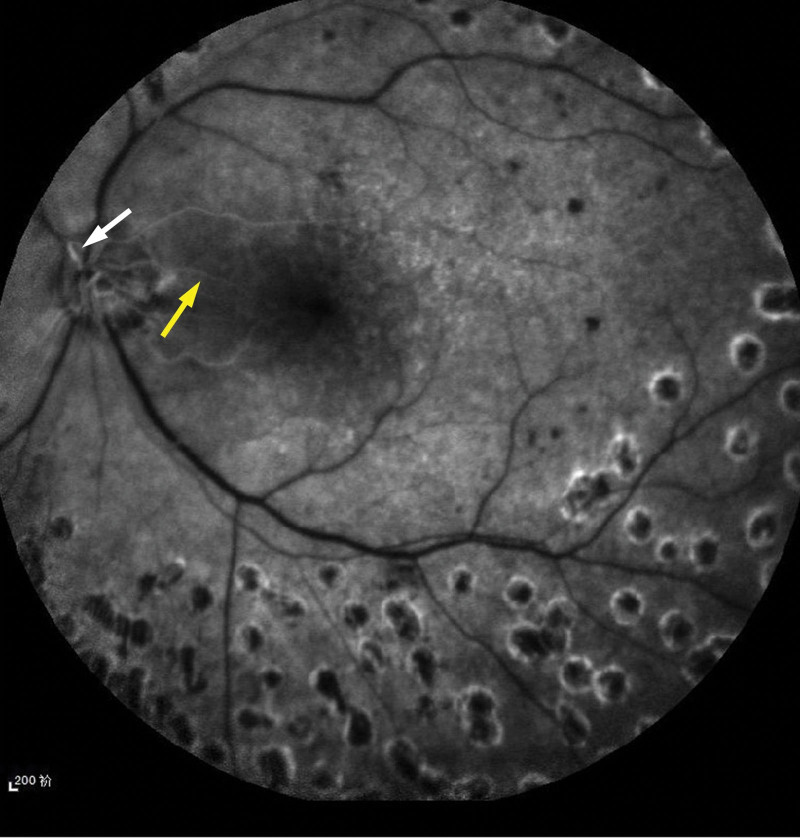
At the patient’s first visit, FFA showed no filling of the central retinal artery at 40 seconds, and the leading edge of the dye (white arrow) was only 1 mm away from the optic disc, suggesting there was retinal circulation insufficiency and that the ciliary reticular artery was supplying part of the macular area (yellow arrow). FFA = fundus fluorescein angiography.

On August 4, 2021, the patient presented with a sudden, painless decline in visual acuity in his left eye. For personal reasons, he did not receive timely intervention and was treated in the ophthalmology department 2 days later. Upon examination, his diabetes had worsened, with an HbA1c level of 12%, random serum glucose of 15.6 mmol/L and BP of 152/96 mm Hg. The best corrected visual acuity (BCVA) of the left eye showed weak light perception, with an intraocular pressure of 14.2 mm Hg. Whole retinal edema was observed in the fundus, and gray retinal opacification was observed in the macular area, but a cherry-red spot was not observed (Fig. 1C, white arrow). OCT revealed retinal edema in the macula, SRF in the papillomacular bundle with a height of 205 μm, nasal macular neurosensory retinal detachment (Fig. 1I, thick arrow), and inner retinal hyperreflectivity (Fig. 1I, thin arrow); the inner retinal thickness nasal to fovea (500 μm away from the foveal center) was 235 μm, and that of the temporal side was 160 μm. Fundus fluorescein angiography (FFA) confirmed that the central retinal artery was not filled at 40 seconds, and the leading edge of the dye was only slightly over 1 mm from the disc (Fig. 2, white arrow), suggesting that the retinal circulation was characterized by hypoperfusion and that the CLRA supplied part of the macula (Fig. 2, yellow arrow).

In view of the treatment delay, the patient was administered conservative therapy, including oxygen inhalation, eye massage, nitroglycerin under the tongue, and Ginkgo biloba extract administered intravenously, but no improvement was observed. Five days later, the left eye was treated with intravitreal injection of ranibizumab. The patient was reexamined 2 weeks later, and his self-reported visual acuity improved (BCVA: HM/20 cm). Funds examination showed reduced whole retinal edema, but macular edema still existed (Fig. 1D). OCT showed reduced macular edema, inner retinal thickness nasal to the fovea (500 μm away from the foveal center) reduced to 202 μm and that of the temporal side reduced to 137 μm, and decreased hyperreflectivity in the inner retina (Fig. 1J, thin arrow), but no obvious changes in SRF (Fig. 1J, thick arrow). The second intravitreal injection of ranibizumab was performed 1 month later, and the patient was reexamined 2 weeks later. The self-reported visual acuity significantly improved. BCVA: 1.4 logMAR units. Fundus examination showed reduced macular edema (Fig. 1E). OCT revealed that the thickness of the inner layer of the nasal side decreased to 133 μm, and that of the temporal side was 107 μm. The hyperreflectivity of the inner retina was significantly reduced (Fig. 1K, thin arrow), but there was still residual SRF (Fig. 1K, thick arrow). Due to domestic COVID-19, the patient did not receive further anti-VEGF treatment and was reexamined after 5 months. Funds examination showed no edema in the whole retina and macula (Fig. 1F). OCT showed that the SRF was completely absorbed, the inner retinal thickness nasal to the fovea became obviously thinner, the retinal thickness nasal to the fovea (500 μm away from the foveal center) was clearly reduced to 127 μm, and the thickness on the temporal side was 98 μm (Fig. 1L). Moreover, BCVA improved to 1.1 logMAR units.

## 3. Discussion

The prevalence of cilioretinal artery-sparing CRAO ranged from 14.0% to 26.0%.^[8]^ The CLRA usually begins in a circular pattern at the base of the optic nerve at the edge of the optic disc, most commonly on the temporal side, and extends towards the macula. The outlet site, number, size, and blood supply area of CLRA may vary from person to person^[9–13]^ and may have different effects on vision.^[14–16]^

Research shows that if there is no cilioretinal macular sparing, there is little hope for visual recovery beyond counting the fingers. If there is sparing of more than half of the papillomacular bundle, but not including the foveola, the visual acuity will range from 6/30 to counting fingers. If cilioretinal foveolar sparing occurs, as in CRAO, eventual visual acuity will be in the range of 6/6 to 6/24.^[7]^ In this case, FFA showed no filling of the central retinal artery at 40 seconds, and the CLRA extended from the temporal side of the optic disc to the macula, supplying more than half of the papillomacular bundle area. OCT showed that, in addition to the inner retinal hyperreflectivity commonly seen in CRAO, SRF was also present in the papillomacular bundle and nasal macular neurosensory retinal detachment.

When CRAO occurs, macular edema in the area of retinal photoreceptor cells may be due to oxygen and energy metabolism failure in the 5 layers of retina cells or excitatory neurotransmitter release of glutamic acid in great quantities from synapses,^[17]^ leading to excessive stimulation of photoreceptor cells through glutamate receptors, producing an excitatory toxin effect^[18]^; furthermore, the function of Mǜller cells in providing nutrition to photoreceptor cells is obviously damaged or lost in hypoxia and energy-metabolism disorders.^[19]^ This patient had undergone surgical procedures, including phacoemulsification and IOL implantation combined with vitrectomy, membrane delamination, and panretinal photocoagulation, for DR complicated by vitreous hemorrhage in the left eye. Studies have shown that intraocular surgery can cause retinal hemodynamic changes and further damage the retinal microcirculation.^[20]^ In addition, the retinal vasculature in the late stage of DR, such as acellular capillaries, microaneurysms, and microvascular lumen stenosis, leads to an increase in hydrostatic pressure in the capillary lumen of the macular area, and the leakage of macromolecules, proteins, and electrolytes with serum into the interstitial space of the macular area leads to SRF in the macular area.^[21,22]^ Knudsen et al^[23]^ also found that the incidence of maculopathy and macular edema increased significantly in diabetic patients with CLRA, which was due to the increased perfusion pressure of the choroid and retina in these patients, causing substances in the choroid to be transported to the macular area, thus aggravating diabetic maculopathy. Studies have shown that PRP treatment leads to generalized narrowing of retinal arterioles and venules.^[24,25]^ Significant constrictions of both retinal arterioles and venules by 10% to 15% after PRP, whereas there was no significant change in the control eye.^[26]^ This may reflect the autoregulatory reduced blood flow subsequently to a lower metabolic demand caused by the partial destruction of retinal tissue, thereby increasing the risk of vascular occlusion. The patient’s vision showed only weak light perception at the onset of the disease. Combined with the patient’s previous medical history and eye surgery history, we believe that although the patient had CLRA, diabetic macular edema and SRF caused by macular capillary leakage might have aggravated the visual impairment.

The cherry-red spot characteristic of the CRAO was not observed in this case. The cherry-red spot is caused by the interruption of blood flow in the central retinal artery during CRAO, resulting in diffuse gray edema at the posterior pole of the retina. In such cases, the macular fovea exhibits a striking cherry-red spot against the surrounding gray edema.^[27]^ Brown et al^[28]^ found that the size of the cherry-red spot varies depending on the width of the fovea. In this case, after the onset of CRAO, the SRF in the papillomacular bundle resulted in neurosensory detachment, which may have masked the cherry red spot. Other studies have shown that a cherry-red spot may not appear if macular ganglion cells and nerve fibers are destroyed.^[29]^ We speculated that in this case, the lack of typical a cherry-red spot may have been due to retinal cell hypoxia, lack of nutrients, and decomposition product accumulation as CRAO occurred, coupled with the existence of CLRA and its breakdown products that were brought to the macular area through its unique vascular access, causing macular edema and SRF in the papillomacular bundle, thus forming atypical a cherry-red spot. In addition, previous damage and disease of the macula may also lead to rapid fading of the early cherry red spot. In conclusion, early diagnosis of CRAO in patients with DR-sparing CLRA who have received prior vitrectomy should be made with caution. A cherry-red spot is not a reliable sign for diagnosing CRAO. OCT cannot be used as a diagnostic indicator because it is of little value in the presence of preexisting diffuse inner retinal atrophy. Therefore, the delayed fill time observed by FFA is still the most reliable validation indicator.

Neurosensory detachment in CRAO patients with CLRA spring has not been previously documented. The present patient was adequately treated for CRAO; however, there was no significant improvement in vision, considering that the longer the CRAO was present, the more severe the retinal edema and the greater the neurosensory detachment. One study indicated that the incidence of choroidal neovascularization (CNV) caused by SRF in the macula is 46%, and this condition can worsen vision loss and other intractable problems.^[30]^ Therefore, to preserve the patient’s vision and avoid more serious eye damage, we tried to apply anti-VEGF therapy with the consent of the patient.

Ranibizumab is a recombinant humanized Fab antibody fragment that blocks human VEGF. Intravitreal injection can effectively reduce retinal capillary leakage and improve retinal microcirculation.^[31,32]^ VEGF stimulates endothelial production of nitric oxide, which is well documented as a vasodilator. Inhibition of VEGF is thought to reduce nitric oxide synthesis and consequently promote vasoconstriction and increase peripheral resistance.^[33–35]^ Studies have shown that eyes with CRVO have variable degrees of ischemia. This causes both platelet activation and the initiation of a coagulation cascade.^[36]^ It has also been reported that the use of anti-VEGF agents to treat macular edema caused by central retinal vein occlusion (CRVO) may increase the risk of retinal artery occlusion.^[37]^ Although CRAO occurred in this case, anti-VEGF therapy may have increased the likelihood of retinal artery occlusion. However, considering the long-term DR, macular edema, and SRF of the patient, which may have aggravated the CNV, FFA showed no obvious retinal artery stenosis. Intravitreal injection of ranibizumab was administered after comprehensive consideration of its advantages and disadvantages. This study aimed to reduce macular edema and prevent neovascular glaucoma by inhibiting CNV. In this case, after 2 intravitreal injections of ranibizumab, the macular edema was reduced, the SRF in the macular area was significantly absorbed, and the visual acuity improved from weak light perception to 1.1 LogMAR units.

In addition, we also noted that the inner retinal nasal-to-fovea thickness decreased and there was significant atrophy 5 months after the second anti-VEGF treatment, which may have been the natural change observed in the course of CRAO^[38]^ or the effect of anti-VEGF treatment. Chatziralli et al^[39]^ found that the significant decrease in central retinal thickness (CRT) was accompanied by improved visual acuity in 110 patients with diabetic macular edema treated with anti-VEGF. Generally, during the natural course of CRAO, retinal atrophy is accompanied by no improvement or a decline in visual acuity. In this case, although the retinal thickness of the macula was significantly thinner, visual acuity was improved and stable at 1.1 logMAR units. The reason for this remains to be further discussed.

## 4. Conclusions

We report the first case of CRAO in a DR patient whose cherry-red spot may have been masked by the presence of CLRA, macular edema, and SRF; consequently, the delayed filling time observed by FFA remains the most reliable marker for CRAO confirmation. For such patients, when CRAO occurs, anti-VEGF therapy can be administered according to the condition to inhibit choroidal neovascularization, reduce macular edema and SRF accumulation, and save some vision. However, there are some limitations in this case, such as the absence of OCTA, the inability to observe the macular microvascular status, and the effect and prognosis of anti-VEGF therapy, which still need to be verified by long-term follow-up and more cases.

## Author contributions

Conceptualization: Ying-Jun Li.

Resources: Fa-Li Jia, Han Zhang.

Software: Yuan Qin, Jing-Hui Liu, Fa-Li Jia, Han Zhang, Jia-Hao Li.

Writing – original draft: Yu-Jie Jia, Hong-Bo Liu.

Writing – review and editing: Yu-Jie Jia, Hong-Bo Liu, and Ying-Jun Li.
